# Analysis of spatial-temporal pattern, dynamic evolution and influencing factors of health tourism development in China

**DOI:** 10.1038/s41598-023-42462-x

**Published:** 2023-09-18

**Authors:** Huadi Wang, Yue Feng, Shaogui Xu, Xiaomei Xu, Kefeng Jiang, Xinyu Nie, Nianxing Zhou

**Affiliations:** 1 School of Education Science, Nanjing Normal University, Nanjing, 210097 China; 2 School of Management, Jinan University, Guangzhou, 510631 China; 3 School of Geography, Nanjing Normal University, Nanjing, 210023 China; 4 Jiangsu Center for Collaborative Innovation in Geographical Information Resource Development and Application, Nanjing, 210023 China

**Keywords:** Socioeconomic scenarios, Sustainability, Health care economics

## Abstract

The evaluation index system is constructed based on the connotation and characteristics of health tourism. Using the entropy method, Thiel index, exploratory spatial data analysis method, spatial Markov chain and spatial econometric model, research is carried out around the development index, difference status, spatial-temporal pattern, dynamic evolution and influencing factors of health tourism. The following results were drawn: (1) The development index of health tourism in China is low, but the development speed is fast. The inter-regional development index shows an eastern China > central China > western China pattern, and the development speed exhibits a western China > central China > eastern China situation. (2) In the overall difference in China’s health tourism development, the intra-regional difference is consistently higher than the inter-regional difference. Among the three major regions, the overall difference between eastern China and western China is always higher than that of central China. (3) The development of health tourism in China is positively correlated in the global space, with some local spatial clustering. (4) The dynamic evolution of health tourism development in China shows part of the “Matthew effect” characteristics, with an obvious spatial spillover effect. (5) Various influencing factors produced widely varying direct, indirect and total effects on health tourism development in China, eastern China, central China and western China. Finally, based on the results of the above empirical analysis, policy recommendations to promote the development of health tourism in China are proposed.

## Introduction

The comprehensive promotion of the Health China strategy has brought new opportunities for the development of health tourism and promoted the increasing maturity of health tourism in China. Health tourism emerged in the late twentieth century, and with the rapid rise of economic and social development, it has now grown into one of the fastest-growing new industries in the global tourism economy. According to the Global Health Institute (GWI), health tourism accounted for $0.639 trillion of the $4.5 trillion global health economy market in 2020, representing 14.2% of the total market and showing a very broad prospect for development^1^. However, the development of health tourism in China has been hindered by an uneven distribution of resources and inefficient supply distribution, causing spatial development imbalances and regional difference that pose obstacles to the comprehensive development of health tourism. China’s provinces urgently need to explore their resources and industrial characteristics. They should rely on their existing advantages in economic, social, cultural, ecological, transportation, and communication aspects to accurately position the development direction, optimize resource integration, and adjust the spatial pattern of the industry. The study of the spatial-temporal pattern of health tourism development, its dynamic evolution and its influencing factors is of great value in clarifying the overall situation of health tourism development in China, identifying the development gaps among provinces, and promoting the comprehensive development of health tourism.

With the COVID-19 pandemic and the increase of tourists’ health awareness, health tourism will show stronger vitality in the post-pandemic era. There are numerous studies on health tourism in China and abroad. Ferrero et al. (2022) conducted a meta-analysis of the literature in the Scopus database and found that tourist behavior and travel motivation of health tourism were the most studied^2^. Zhong et al. (2023) conducted a comprehensive review of 40 years of literature in the Chinese CNKI database and classified Chinese health tourism studies into different stages, namely “exploration stage of health tourism (1981–2001)”, “initial stage of health tourism (2002–2012)”, “development stage of health tourism driven by demand (2013–2015)”, and “growing stage of health tourism driven by policy (2016–2020)”. The author also identified “wellness tourism”, “health and wellness tourism”, and “medical tourism” as the main areas of focus, and pointed out that China’s health tourism is evolving towards industrialization, diversification, and systematization^3^. Overall, studies on health tourism in China and abroad mainly focus on the connotation, value, and development of health tourism. However, due to the difference in defining the concept of “health tourism”, therefore, the study conducted with terms such as “wellness tourism”^4,5^ and “medical and health tourism”^6^ also fall within the scope discussion of this paper. To address this partial disagreement in understanding, some studies have focused on clarifying the characteristics^7,8^ and historical development^9^ of health tourism. Some specific studies in China and abroad have also covered various aspects of health tourism. In terms of study content, for the better development of health tourism, the effective use of resources^10^ and product development^11,12^ have been focused. Meanwhile, some studies have investigated the current status of development^13–16^, focusing on the influencing factors behind it^17–19^ and proposing corresponding countermeasures and suggestions^20–24^. In addition, the latest studies have linked the development of health tourism to the COVID-19 pandemic and predicted the willingness to health travel after the COVID-19 pandemic^25^. In terms of study perspective, some studies have sought to understand the reality and consumer demand for health tourism in China^26^, and introduced a participant network perspective to study the synergistic evolution and internal vitality of healing landscapes and health tourism^27^. The studies have also explored the effect of health tourism on recovery place attachment^28^. Finally, the issue of dynamic systems and mechanisms of health tourism innovation development^29^ has also been a concern to a certain extent. With the continuous improvement of health tourism in tourism development, promoting the comprehensive development of health tourism among provinces and enhancing its overall competitiveness in China has become an important study topic in the context of high-quality development of tourism. Previous studies on health tourism have laid a good foundation for this study, but there are still several issues worth further discussion. Firstly, although existing studies have analyzed the current situation, factors, and countermeasures of health tourism development, most of them have been conducted from a micro perspective and have failed to present the spatial pattern and dynamic evolution of health tourism development. Secondly, most of the existing studies adopt a general analytical approach^30,31^, the lack of empirical data prevents us from assessing the dynamics of health tourism and the relationship between regions in the overall space. Methods such as the Thiel index, exploratory spatial technology analysis, and spatial Markov chain can provide a comprehensive understanding of spatial changes, thereby making the study’s conclusions more reliable. Thirdly, the study results mostly focus on the analysis of the current situation and proposed countermeasures based on a general and descriptive evaluation of the development status of health tourism. This approach not only lacks scientific and reasonable evaluation and analysis but also suffers from a noticeable lack of practicality and relevance in the proposed countermeasures, which needs further study and development.

In summary, this study aims to establish a scientific and reasonable evaluation index system for health tourism based on relevant literature and books. It will measure the comprehensive development index by assigning weights using the entropy method, and calculate the difference status of development using the Thiel index. In addition, the study will employ the exploratory spatial data analysis method (ESDA) and spatial Markov chain to comprehensively understand the spatial and temporal pattern, dynamic evolution, and spatial association of health tourism in China. Then, the influencing factors of health tourism development were analyzed through the spatial econometric model. Finally, based on the above empirical analysis policy recommendations to promote the development of health tourism are proposed. The goal is to gain a comprehensive understanding of the spatial and temporal characteristics of health tourism development in China and its influencing factors, as well as to positively influence the recovery and high-quality development of the health tourism industry and the tourism industry in the post-epidemic era.

## Methodology and data

### Research methodology

#### Entropy Method

The entropy method is an objective assignment method that can effectively avoid the subjectivity of indicator assignment. The calculation steps of the entropy method are mainly referred to in the study of Guo et al. (2019)^32^. The final equation is as follows:

1 $$y_{i} = \sum\limits_{j = 1}^{{\text{n}}} {w_{j} \times x_{ij}^{*} }$$

In the equation,$y_{i}$ denotes the composite score of the* i*th object, i = 1,2, ……, 31.$w_{j}$ denotes the weight of the *j*th *indicator*, and $x_{ij}^{*}$ denotes the standardized value of the *j*th indicator of the* i*th object.

#### Thiel index

The Theil index is a useful tool for comparing the differences in health tourism development index within different regional systems, as it is not affected by the number of spatial units examined^33^. The Theil index can be decomposed into intra-regional and inter-regional variation to better analyze the regional variability^34^. Moreover, the size of the Thiel index has a positive relationship with the variability of health tourism development, i.e., the larger the numerical value, the greater the variability, and the smaller the numerical value, the smaller the variability, and its numerical value range are [0,1]^35^. The equation for calculating the Thiel index is as follows^36^:

2 $$T_{t} = \frac{1}{n}\sum\nolimits_{i = 1}^{n} {(Y_{it} Y_{t} )log\left( {\frac{{Y_{it} }}{{Y_{t} }}} \right)}$$

3 $$T_{j,t} = \frac{1}{{n_{j} }}\sum\nolimits_{i = 1}^{{n_{j} }} {(Y_{ijt} Y_{jt} )log\left( {\frac{{Y_{ijt} }}{{Y_{jt} }}} \right)}$$

4 $$T_{t} = TWR + TBR = \sum\nolimits_{j = 1}^{3} {\left( {\frac{{n_{j} }}{n} \times \frac{{Y_{j,t} }}{{Y_{t} }} \times T_{j,t} } \right)} + \sum\nolimits_{j = 1}^{3} {\left( {\frac{{n_{j} }}{n} \times \frac{{Y_{j,t} }}{{Y_{t} }} \times \log \frac{{Y_{j,t} }}{{Y_{t} }}} \right)}$$

In Eq. (2), *T*_*t*_ represents the health tourism development index of China. In Eq. (3), *T*_*j,t*_ denotes the overall difference in the Thiel index of health tourism development index in the three major regions (*j* = 1, 2, 3) in eastern China, central China and western China, respectively. *n* represents the total number of provinces in China, and *n*_*j*_ denotes the number of provinces in the three major regions in eastern China, central China and western China, respectively. *y*_*i,t*_ represents the health tourism development index of province *i*, and *y*_*i,j,t*_ denotes the health tourism index of province *i* in region *j. y*_*t*_ and *y*_*j,t*_ represent the average value of the health tourism development index in China and the average value of the health tourism development index in region *j*. In Eq. (4), *TWR* and *TBR* represent the intra-regional difference Theil index and inter-regional difference Theil index after decomposition of the total Theil index *T*_*t*_, respectively.

#### Exploratory spatial data analysis (ESDA)

Exploratory Spatial Data Analysis (ESDA) is a method of analyzing spatial data using statistical principles, graphical images, and other methods to detect non-randomness or spatial autocorrelation in spatial distributions^37^. According to the different functions, spatial correlation is divided into global spatial correlation and local spatial correlation, which can effectively reveal the spatial clustering phenomenon and characteristics of the health tourism development index in each province of China.

(1) Global spatial autocorrelation

The global spatial autocorrelation uses Moran’s *I* index to reflect the similarity, dissimilarity, and association deconstruction patterns of the elements in the entire study area^38^, which is calculated by the equation:

5 $$I = \frac{N}{{\sum\nolimits_{i} {\sum\nolimits_{j} {W_{ij} } } }}\frac{{\sum\nolimits_{i} {\sum\nolimits_{j} {W_{ij} (X_{i} - \overline{X})(X_{j} - \overline{X})} } }}{{\sum\nolimits_{i} {(X_{i} - \overline{X})^{2} } }}$$

In the equation, *N* = 31 denotes 31 provinces in China; *X*_*i*_ denotes the health tourism development index of the* i*th province and region, and $\overline{x }$ denotes the average value of the health tourism development index of 31 provinces, *ω*_*ij*_ indicates the spatial weight. Moran’*s I* take a value range of [− 1,1], when the value is close to 1, there is a positive correlation and a cluster distribution pattern; when the value is close to − 1, there is a negative correlation and a discrete distribution pattern; when Moran’*s I* = 0, it means no correlation and a random distribution pattern^39^.

(2) Local spatial autocorrelation

The correlation index of local spatial autocorrelation can reflect the correlation of local health tourism development, but also can reveal the spatial distribution pattern of high-value clusters and low-value clusters, i.e. hot spot and cold spot areas in different spatial locations^40^. The calculation equation is as follows:

6 $$I_{i} = Z_{i} \sum\limits_{i}^{n} {W_{ij} Z_{j} }$$

7 $$Z_{i} = (X_{i} - \overline{X})/\sqrt {\frac{1}{n}\sum\limits_{i = 1}^{n} {(X_{i} - \overline{X})^{2} } }$$

In the two equations, *Z*_*i*_ and *Z*_*j*_ are the attribute values (development index) of the studied provinces *i* and *j*, respectively, and *ωij* is the spatial weight matrix. *Ii* is the local correlation index of unit *i*, indicating the degree of correlation with other regions^38^.

(3) Spatial weight matrix

The spatial weight matrix plays an important role in spatial analysis, as geographical proximity and economic association are crucial factors affecting the spatial layout of economic activities. The spatial association between regions is most likely to come from the dual influence of geographical proximity and economic association. Based on this, the gravity model is chosen to construct a spatial weight matrix that integrally reflects the geographic and economic distance ^41^*. W*_*ij*_ is the spatial weight matrix, and the calculation equation after adopting the gravitational model weight matrix is

8 $$W_{gav} = \tau W_{dis} + (1 - \tau )W_{eco}$$

In the equation, *τ* takes the value of 0.5, and the equation of the geographic adjacency matrix *W*_*dis*_ is

9 $$W_{dis} = {1 \mathord{\left/ {\vphantom {1 {d_{jv}^{2} }}} \right. \kern-0pt} {d_{jv}^{2} }},\;\;\;j \ne v$$

In the equation, *d*_*jv*_ is the distance between provinces calculated using latitude and longitude data and *j* ≠ v; when *j* = *v*, then the result is 0, and 2 is the geographic attenuation parameter.

The spatial weight matrix of economic adjacency *W*_*eco*_ is constructed with GDP per capita, and its equation is

10 $$W_{eco} { = }{1 \mathord{\left/ {\vphantom {1 {\left| {\overline{Q}_{j} - \overline{Q}_{v} } \right|}}} \right. \kern-0pt} {\left| {\overline{Q}_{j} - \overline{Q}_{v} } \right|}},\quad j \ne v$$

In the equation, *j* ≠ v, $\overline{Q}_{j}$ is the average value of GDP per capita in province *j* from 2010 to 2019.

#### Spatial markov chain

The traditional Markov chain is a method for studying stochastic transfer problems with discrete time and state under no posterior conditions^42^. The spatial Markov chain method is the product of combining the traditional Markov method with the concept of spatial autocorrelation or spatial lag^43^, which compensates for the neglect of the spatial correlation of health tourism development between regions by the traditional Markov chain. The spatial Markov chain transfer probability matrix is conditioned on the spatial lag of a region *i* in the initial year and divided into N types as well, grading the traditional N × N Markov transfer matrix into N N × N transfer conditional probability matrices. The matrix *P*_*Ni/j*_ denotes the spatial transfer probability of transferring from state* i* to state j at *t* + 1 when the spatial lag type is N in year t. For region *i*, whose neighborhood is *j*, the spatial lag of location *i* is calculated as

11 $$Lag_{i} = \sum\limits_{j = 1}^{n} {Y_{j} W_{ij} }$$

In the equation, *Y*_*j*_ is a province's health tourism development index; *n* is the total number of provinces; *W*_*ij*_ is the spatial weight matrix.

#### Spatial econometric model

Spatial panel models can effectively solve the problems of spatially explained variable autocorrelation and measurement error and mainly include the spatial autoregressive model (SAR), spatial error model (SEM) and spatial Durbin model (SDM)^44^. Considering the possible spatial spillover effects between the influencing factors of health tourism development in each province in China, based on the basic spatial panel model, the specific model is constructed as follows:

12 $$\begin{aligned} TD_{it} = &\beta_{0} + \beta_{1} \ln ES_{it} + \beta_{2} SS_{it} + \beta_{3} \ln TC_{it} + \beta_{4} \ln TS_{it} \hfill \\ &+ \beta_{5} IS_{it} + \beta_{6} \ln IB_{it} + \beta_{7} CD_{it} + \beta_{8} \ln ED_{it} + \mu_{i} + \varepsilon_{it} \hfill \\ \end{aligned}$$

In the equation, *i* is the province, *t* is the year, and *TD*_*it*_ is the health tourism development index. ln*ES*_*it*_ is the economic situation; *SS*_*it*_ is the social situation; ln*TC*_*it*_ is the traffic condition; ln*TS*_*it*_ is the talent situation; *IS*_*it*_ is the industrial structure; ln*IB*_*it*_ is the industrial base; *CD*_*it*_ is the consumer demand; ln*ED*_*it*_ is the employment demand; *μ*_*i*_ is the individual fixed effect and *ε*_*it*_ is the error term.

In addition, given the possible omission of location factors and other variables in the setting of the econometric model, these unobservable missing variables may also have an impact on the health tourism development index and lead to spatial dependence, so it is necessary to include the spatial effect in the econometric analysis. The specific spatial econometric model is set as follows:

13 $$\begin{gathered} y_{it} = \alpha + \rho \sum\limits_{j = 1,j \ne i}^{N} {W_{ij} y_{it} } + \beta X_{it} + \sum\limits_{j = 1,j \ne i}^{N} {W_{ij} X_{ijt} \theta } + \mu_{i} + \varepsilon_{it} \hfill \\ \varepsilon_{it} = \varphi \sum\limits_{j = 1,j \ne i}^{N} {W_{ij} \varepsilon_{jt} } + \phi_{it} \hfill \\ \end{gathered}$$

In the equation, ε_*it*_ is the error term; *μ*_*i*_ is the unobservable regional effect; *ρ* and *φ* are the spatial lag coefficient and spatial error coefficient, respectively; *W*_*ij*_ is the spatial weight matrix; and *X* is the independent variable vector including the economic situation, social situation, traffic condition, talent situation, consumer demand and employment demand. Equation (13) is a general nested model of the spatial interaction effect^45^. In the empirical analysis, according to *ρ*, *φ* and *θ*, the spatial econometric model is also different depending on whether the value is 0.

### Index system construction

**Figure 1:**
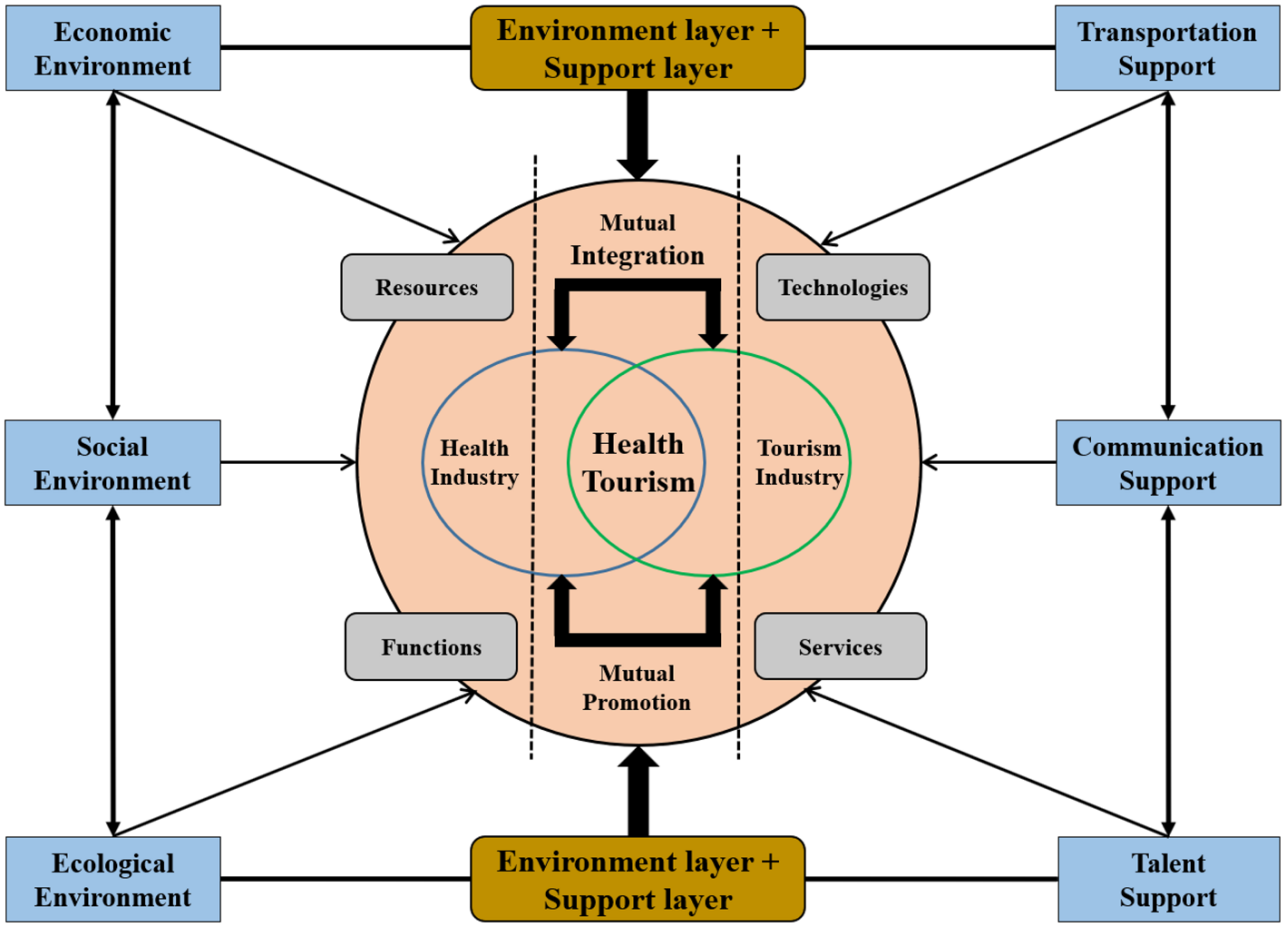
Conceptual framework for evaluating health tourism development.

Constructing a scientific and reasonable evaluation index system is crucial as it serves as the premise and important foundation for accurately measuring the health tourism development index. Regarding the definition of health tourism, the “Health China 2030 Planning Outline” plan issued in October 2016 describes it as “a new industry that integrates health services and tourism”. Jin & Wang (2020) believe that health tourism is a product of the integration of health and tourism, and its development is essentially the integration process of health industry and tourism industry^46^. Yang &Shi (2020) argue that health tourism refers to a new form of integrated tourism that is gradually formed by the health service industry and the tourism industry through resource sharing, function extension, technology and service penetration, mutual penetration and extension to enhance health benefits^23^. Liu et al. (2023) believe that health tourism is a new tourism mode combining tourism industry and health industry, which can meet people’s demand for improving life quality and physical and mental health^47^. In summary, this study defines health tourism as “a new form of integrated tourism industry combining health and tourism”. From national documents and relevant studies, health services and tourism supply and the integration of the two are key to assessing the basis for the development of health tourism. Therefore, the “development foundation” subsystem of the evaluation index system constructed in this study includes the two major components of the health industry and the tourism industry, as well as some integration indicators. In addition, external factors affecting the integrated development of health tourism are also fully considered in this study. Li and Chen (2021) constructed a system of motivating factors to assess the development of health tourism from four aspects: market demand, industry supply, infrastructure and external environment^48^. Yang &Xia (2022) argue that the intrinsic mechanism of the integration and development of the tourism industry and the health industry is driven by external factors such as market demand, macro policies and technological innovation ^49^. Yang &Shi (2020) believe that the integration of tourism industry and health industry will be stimulated by the co-stimulation of the external environment of the industry and the internal environment of the industry to form an environmental layer of integrated development^23^. Therefore, this study also tries to construct a health tourism development evaluation index system with internal and external interaction. Based on relevant literature, this study includes economic, ecological and social environmental factors that directly affect the development of health tourism^50,51^ and external support such as transportation, communication and talent that indirectly affect the development of health tourism^52,53^. The comprehensive health tourism evaluation index system of “environment–foundation–support” coordination and “external–internal–external” interaction is constructed, which is no longer limited to a single evaluation of the actual situation of health industry and tourism industry itself (Fig. 1).

In the whole health tourism evaluation index system, the development environment such as economy, ecology and society and the development support such as transportation, communication and talents together construct the external elements to stimulate the integrated development of health tourism, and the industrial composition, industrial scale and industrial level together constitute the internal foundation of health tourism development. After constructing the evaluation index system, this study adopts the entropy value method to determine the weights of 42 indicators used to evaluate the health tourism development index (Table 1).

**Table 1:** Health tourism development evaluation index system and weights.

Target layer	Subsystems	Guideline layer		Indicator layer	Positive and negative	Weights
Health tourism development index	Development environment	Economic environment		GDP per capita	+	0.012
				Total retail sales of social consumer goods per capita	+	0.033
				Total Import and Export	+	0.087
				The average annual wage of active employees	+	0.006
		Ecological environment		Parkland area per capita	+	0.002
				Water resources per capita	+	0.180
				Public toilets per 10,000 people	+	0.006
				Harmless treatment rate of domestic waste	+	0.001
				Greening coverage of built-up areas	+	0.001
				Forest cover	+	0.015
		Social environment		Population density	+	0.008
				Population urbanization rate	+	0.003
				Pension insurance participation rate	+	0.013
				Basic medical insurance participation rate	+	0.012
	Development foundation	Industry composition	Tourism industry	Number of star-rated hotels	+	0.013
				Number of tourist agencies	+	0.020
				Number of A-class scenic spots	+	0.022
			Health industry	Number of medical and health institutions	+	0.023
			Integration indicators	Number of legal entities in the accommodation and catering industry	+	0.034
				Number of legal entities in the health, social security and social welfare sector	+	0.029
				Number of legal entities in culture, sports and entertainment	+	0.042
		Industry Scale	Tourism industry	Domestic tourism revenue	+	0.032
				Foreign exchange tourism revenue	+	0.065
				Number of domestic tourists	+	0.030
				Number of inbound tourists	+	0.038
			Health industry	Number of health personnel	+	0.020
			Integration indicators	Employed persons in accommodation, food and beverage units	+	0.035
				Employed persons in cultural, sports and entertainment units	+	0.020
				Employed persons in health and social work units	+	0.018
		Industry level	Tourism industry	Domestic tourism revenue as a share of GDP	+	0.012
			Health industry	Number of health technicians per 10,000 persons	+	0.004
				Number of practicing (assistant) physicians per 10,000 persons	+	0.004
				Number of registered nurses per 10,000 persons	+	0.003
				Number of beds in medical institutions per 10,000 persons	+	0.006
			Integration indicator	Employees in the tertiary industry as a share of total employed persons	+	0.002
	Development support	Transportation support		Railroad operating mileage	+	0.017
				Urban road area per capita	+	0.005
				High-grade road mileage	+	0.015
		Communication support		Cell phone penetration rate	+	0.003
				Internet penetration rate	+	0.004
				Total postal business	+	0.102
		Talent support		Average number of students enrolled in higher education per 100,000 persons	+	0.005

### Data source

This study selected 31 provinces in the study area (excluding Hong Kong, Macao, and Taiwan). Each province was used as the basic spatial unit for analysis, and the relevant study was conducted at three regional spatial scales: eastern China, western China, and central China, as well as the national spatial scale. The data used in the study were obtained from various sources, including the China Statistical Yearbook, China Agricultural Yearbook, China Environmental Statistical Yearbook, China Urban and Rural Construction Statistical Yearbook, and statistical bulletins of each province from 2011 to 2020. To ensure the data meet the requirements of authenticity, completeness, and continuity, the missing data from some years in individual provinces were supplemented by using linear processing.

## Empirical results

### Measurement of development index

Table 2 reports the development index and rate of increase in health tourism for the whole country, three major regions, and 31 provinces from 2010 to 2019. First, from the overall national situation, the health tourism development index improved from 0.12 in 2010 to 0.23 in 2019, with an increased rate of 7.67%, which is a fast development rate and reflects the rising trend of health tourism development in China. The reasons for this: the rapid economic and social development and the rapid rise of middle-income class people stimulate people’s potential consumer demand for health tourism and enhance their invisible consumption ability, and tourism consumption is undergoing structural changes in the process of transformation and upgrading. However, judging from the current development index (0.12–0.23), health tourism still has much room for development, and it is expected to develop further as people’s health awareness continues to increase and government support for health tourism continues to grow. Secondly, from the perspective of the three regions, the average development index and development speed of health tourism are not consistent. In terms of the development index, the development index of eastern China (0.23) is higher than that of central China (0.15), and the development index of central China is higher than that of western China (0.13). The top five provinces in the health tourism development index are all located in eastern China, and four of the bottom five provinces are located in western China. For a long time, eastern China has been the center of gravity of China’s economic development, with great advantages in technology and capital. Although the abundance of resources in central China is higher than that in eastern China, there is still a big gap between the economic and social development of central China and eastern China. Although western China has obvious advantages in resources, the relative backwardness of transportation, communication and other infrastructures largely limits the development level of health tourism. In terms of growth rate, eastern China (6.75%) is lower than central China (7.34%), and central China is lower than western China (8.74%), which reflects the momentum of catching up with relatively advanced regions in the backward area of health tourism development. With the implementation of strategies such as the development of western China and the rise of central China, as well as the decline in the influence of factors such as technology and capital and the increase in the influence of factors such as resources and environment, the development advantages of western China and central China have gradually emerged, and the growth rate has accelerated significantly.

**Table 2:** Health tourism development index and ranking in China from 2010 to 2019.

Provinces	Year										Average value	Average ranking	Growth rate	Growth ranking
	2010	2011	2012	2013	2014	2015	2016	2017	2018	2019				
Beijing	0.21	0.23	0.24	0.26	0.26	0.26	0.27	0.28	0.33	0.33	0.27	5	5.24%	27
Tianjin	0.08	0.09	0.10	0.10	0.11	0.11	0.12	0.13	0.12	0.13	0.11	25	5.10%	28
Hebei	0.12	0.13	0.14	0.15	0.16	0.17	0.19	0.20	0.23	0.25	0.17	14	7.93%	14
Liaoning	0.14	0.15	0.17	0.18	0.18	0.17	0.18	0.18	0.20	0.20	0.18	13	3.86%	30
Shanghai	0.18	0.19	0.19	0.20	0.21	0.21	0.23	0.24	0.27	0.28	0.22	6	5.23%	26
Jiangsu	0.22	0.24	0.26	0.26	0.28	0.30	0.32	0.36	0.39	0.42	0.30	2	7.52%	18
Zhejiang	0.20	0.22	0.23	0.26	0.28	0.30	0.32	0.36	0.37	0.41	0.29	3	8.36%	12
Fujian	0.13	0.14	0.15	0.16	0.18	0.19	0.20	0.23	0.25	0.28	0.19	10	9.18%	8
Shandong	0.20	0.22	0.23	0.26	0.27	0.28	0.30	0.33	0.35	0.37	0.28	4	7.09%	21
Guangdong	0.31	0.34	0.37	0.41	0.43	0.45	0.47	0.52	0.60	0.65	0.45	1	8.68%	10
Hainan	0.06	0.06	0.07	0.07	0.07	0.07	0.08	0.08	0.09	0.10	0.07	29	6.01%	24
The average of eastern China	0.17	0.18	0.19	0.21	0.22	0.23	0.24	0.26	0.29	0.31	0.23	(1)	6.75%	(3)
Shanxi	0.09	0.10	0.11	0.11	0.12	0.12	0.13	0.15	0.16	0.17	0.13	21	7.65%	17
Jilin	0.08	0.09	0.09	0.10	0.10	0.11	0.11	0.12	0.13	0.13	0.11	26	5.78%	25
Heilongjiang	0.10	0.11	0.12	0.12	0.12	0.12	0.12	0.14	0.14	0.15	0.12	22	3.82%	29
Anhui	0.09	0.10	0.11	0.13	0.14	0.15	0.17	0.18	0.21	0.23	0.15	17	10.31%	3
Jiangxi	0.09	0.10	0.11	0.12	0.13	0.14	0.15	0.17	0.18	0.20	0.14	19	8.53%	11
Henan	0.16	0.16	0.17	0.20	0.21	0.23	0.23	0.25	0.27	0.29	0.22	7	6.97%	23
Hubei	0.13	0.13	0.15	0.16	0.17	0.19	0.20	0.22	0.23	0.24	0.18	11	7.63%	16
Hunan	0.13	0.13	0.14	0.16	0.17	0.18	0.19	0.21	0.23	0.25	0.18	12	8.02%	13
The average of central China	0.11	0.12	0.13	0.14	0.15	0.15	0.16	0.18	0.19	0.21	0.15	(2)	7.34%	(2)
Inner Mongolia	0.09	0.09	0.10	0.11	0.12	0.13	0.13	0.15	0.16	0.16	0.12	23	7.46%	19
Guangxi	0.09	0.10	0.11	0.12	0.13	0.14	0.15	0.17	0.19	0.22	0.14	18	9.96%	6
Chongqing	0.08	0.09	0.10	0.12	0.13	0.14	0.15	0.17	0.18	0.19	0.13	20	10.80%	2
Sichuan	0.16	0.14	0.16	0.18	0.20	0.21	0.22	0.25	0.27	0.29	0.21	8	7.32%	22
Guizhou	0.06	0.07	0.08	0.09	0.10	0.11	0.12	0.15	0.17	0.19	0.11	24	13.80%	1
Yunnan	0.10	0.10	0.11	0.13	0.14	0.15	0.17	0.20	0.21	0.23	0.15	16	10.16%	4
Tibet	0.20	0.19	0.18	0.19	0.19	0.22	0.20	0.21	0.21	0.21	0.20	9	0.89%	31
Shanxi	0.11	0.12	0.13	0.15	0.15	0.16	0.17	0.19	0.21	0.22	0.16	15	7.88%	15
Gansu	0.05	0.06	0.06	0.07	0.08	0.08	0.09	0.10	0.11	0.12	0.08	28	9.17%	9
Qinghai	0.04	0.05	0.05	0.05	0.06	0.06	0.06	0.08	0.09	0.09	0.06	30	10.27%	5
Ningxia	0.03	0.03	0.04	0.05	0.05	0.05	0.06	0.06	0.06	0.07	0.05	31	10.04%	7
Xinjiang	0.07	0.08	0.08	0.09	0.09	0.10	0.10	0.11	0.12	0.13	0.10	27	7.16%	20
The average of western China	0.09	0.09	0.10	0.11	0.12	0.13	0.14	0.15	0.17	0.18	0.13	(3)	8.74%	(1)
The average for all of China	0.12	0.13	0.14	0.15	0.16	0.17	0.18	0.20	0.22	0.23	0.17	–	7.67%	–

### Difference in development index

The previous content has analyzed the development index of health tourism at different spatial scales in China as a whole, three major regions, and 31 provinces from 2010 to 2019. However, it was not possible to identify the difference between the development of health tourism across China as a whole and the three largest regions from the statistical data results, and it was also challenging to accurately determine the direction of further in-depth study after measuring and analyzing. Therefore, with the help of the Theil Index, this paper calculates the difference in the health tourism development index and presents it visually. It mainly calculates the overall difference index of China, the difference index of intra-region and inter-region, and the overall difference index of eastern China, central China, and western China, to prepare for future research based on the measurement results of development difference. The summary results are summarized in Table 3.

**Table 3:** Theil index of China health tourism development from 2010 to 2019.

Year	Thiel index of three regions in China			Thiel index of China		
	Theil index of eastern China	Theil index of central China	Theil index of western China	Theil index of intra-regional difference	Theil index of inter-regional difference	Theil index of overall difference
2010	0.084	0.028	0.128	0.087	0.040	0.127
2011	0.088	0.017	0.094	0.076	0.046	0.122
2012	0.084	0.019	0.082	0.071	0.045	0.116
2013	0.093	0.025	0.078	0.075	0.039	0.115
2014	0.092	0.026	0.072	0.073	0.038	0.111
2015	0.098	0.031	0.080	0.080	0.033	0.112
2016	0.091	0.030	0.070	0.073	0.035	0.109
2017	0.101	0.026	0.067	0.076	0.031	0.106
2018	0.108	0.028	0.065	0.080	0.033	0.113
2019	0.110	0.031	0.065	0.081	0.033	0.114

**Figure 2:**
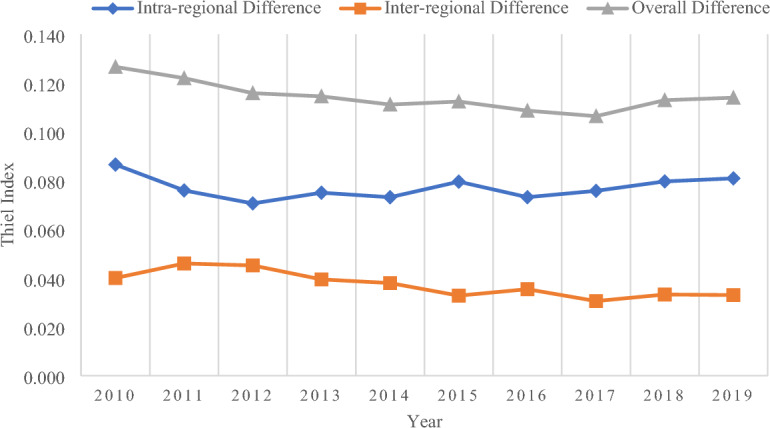
Theil index of China.

Firstly, when looking at China as a whole, the intra-regional difference is always greater than the level of inter-regional difference and maintains a relatively stable development despite fluctuations (Fig. 2). For instance, in 2010 and 2019, the difference between intra-regional and inter-regional was 0.046 and 0.048, respectively. Meanwhile, the intra-regional difference in 2010 was 0.087, accounting for 68.36%, and the inter-regional difference was 0.040, accounting for 31.64%. In 2019, the intra-regional difference was 0.081, accounting for 70.99%, and the inter-regional difference was 0.033, accounting for 29.01%. Based on the above data results, it can be seen that the intra-regional difference occupies a dominant position in the whole study period and has a weak strengthening trend to the end of the investigation, while the influence of inter-regional difference is somewhat weakened. With the rapid development of health tourism, eastern China has strengthened the transformation and upgrading of its industrial structure mainly based on its economic advantages, western China has strengthened the utilization of its good natural foundation and abundant resources, and central China has strengthened its interaction and cooperation with the eastern China and western China, which has made the impact of inter-regional difference among eastern China, central China, and western China become smaller. At the same time, the impact of intra-regional differences plays a major role due to the similarity of social environment, economic situation and resource base within each region of China, which is more likely to be affected by other factors such as policies of provinces and municipalities. In addition, from 2010 to 2012, the change trend of the overall difference was consistent with the intra-regional difference, while the change trend of the overall difference was opposite to the inter-regional difference, and after 2012, the change trend of the overall difference was generally consistent with the inter-regional difference and opposite to the intra-regional difference. Before 2012, China and its relevant departments still paid limited attention to health tourism, and the policy support was relatively low, which did not form a strong development momentum and joint force. The Ministry of Health issued the *“Health China 2020” strategic research report* in 2012, and the State Council proposed the *“Opinions on Promoting the Development of Health Services”* in 2013, and the release of these “policy signals” is of great benefit to the development of health tourism. In 2016, documents such as the *“Health China 2030 Planning Outline”*, the *"Notice of the State Council on Issuing the “Thirteenth Five-Year Health and Health Plan”* and the *Standard of National Health Tourism Demonstration Base* have become important “catalysts” for the development of health tourism. Overall, the development trend of health tourism is good.

**Figure 3:**
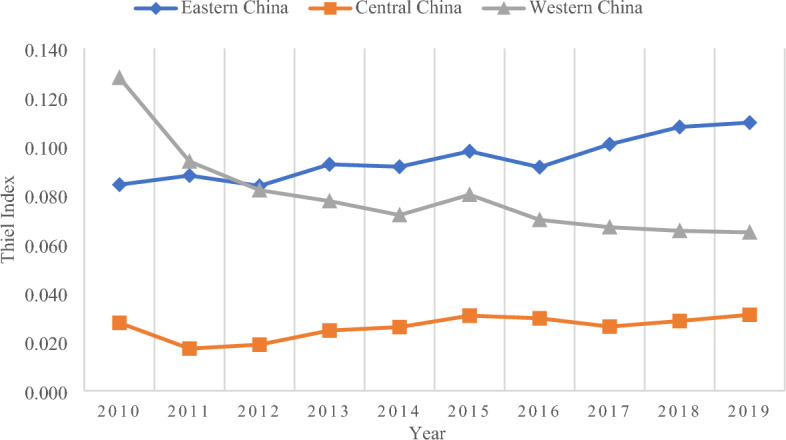
Thiel index of three regions in China.

Comparing the overall Theil Index at the national spatial level only provides the most comprehensive macro-view of health tourism but does not enable further analysis of the actual situation in the area. Therefore, it is necessary to interpret the spatial scale difference between the three regions. From the comparison of the three regions (Fig. 3), it can be found that the overall difference between eastern China and western China is significantly higher than that of central China. In terms of the specific magnitude of difference, the overall difference in western China was greater than that in eastern China before 2012, after which the difference in eastern China began to be greater than that in western China and continued until 2019, and the difference between the overall difference between eastern China and western China increased, the difference between western China and central China decreased, and central China was the region with the smallest overall difference in all the study periods. Finally, the overall difference between Eastern China and Central China both tends to increase and is more significant in Eastern China, while the overall difference in Western China tends to decrease significantly. The transfer of capital and technology from eastern China and the severe lag in economic development in western China have caused the overall difference between the two regions to be higher than that of central China. In addition, the overall difference between eastern China and central China has increased with the decline of economic and technological elements in health tourism development, while the implementation of *western China development strategy* and the full exploitation of quality health tourism resources has led to a reduction in the overall difference in western China.

The above analysis shows that intra-regional difference has always been the dominant factor in the overall difference of health tourism development in China during the study period, while the influence of inter-regional difference is relatively small and the degree of the effect of intra-regional difference tends to increase, while the influence of inter-regional difference decreases, but the overall change is not significant. In terms of different regions, the overall difference between eastern China and central China tends to increase, we should pay attention to this phenomenon, investigate the causes, and create effective measures to reduce the level of difference in the development of health tourism and realize the comprehensive development of health tourism in China.

### Spatial-temporal pattern

The test of Moran’s *I* index shows that the spatial correlation of health tourism development in China is significant (Table 4), and the development of health tourism in each province of China displays a state of agglomeration in space.

**Table 4:** Global autocorrelation test for China’s health tourism development index from 2010 to 2019.

Year	2010	2011	2012	2013	2014	2015	2016	2017	2018	2019
Moran’s *I*	0.097*	0.102*	0.108*	0.103*	0.119*	0.106*	0.110*	0.118*	0.100*	0.098*
Z-value	1.334	1.413	1.485	1.441	1.617	1.469	1.518	1.608	1.442	1.429
*P*-value	0.091	0.079	0.069	0.075	0.053	0.071	0.064	0.054	0.075	0.077

***, **, and * indicate significance at the 1%, 5%, and 10% levels, respectively.

The global Moran’s *I* index identified a positive correlation of the development of health tourism above the global space, but it is unclear how each province’s above-spatial correlation pattern should be interpreted. It is necessary to interpret local autocorrelation status to better comprehend the spatial correlation pattern and spatiotemporal pattern of the development of health tourism. Through the calculation of local autocorrelation, a Moran scatter diagram can be drawn and the clustering characteristics of health tourism development can be described spatially through the LISA diagram. The four quadrants of the Moran scatter diagram represent different spatial management patterns, with high–high (H–H) and low–low (L–L) clustering in the first and third quadrants, respectively, and there is a positive spatial correlation between the provinces in these two quadrants; the second and fourth quadrants are low–high (L–H) agglomeration and high-low (H–L) agglomeration, respectively, and there is a negative correlation between the provinces in these two quadrants^54^. Through sorting out the scatter chart, the local spatial autocorrelation types of health tourism development in China’s provinces during 2010–2019 are summarized in Table 5.

**Table 5:** Types of local spatial autocorrelation of health tourism development index by provinces in China (some years).

Year	First quadrant (HH)	Second quadrant (LH)	Third quadrant (LL)	Fourth quadrant (HL)
2010	Beijing, Shanghai, Jiangsu, Zhejiang, Fujian, Henan, Guangdong, Sichuan (8)	Liaoning, Shandong, Hubei, Hunan, Tibet (5)	Hebei, Shanxi, Jilin, Anhui, Jiangxi, Guangxi, Hainan, Guizhou, Shaanxi, Gansu, Qinghai, Ningxia, Xinjiang (13)	Tianjin, Inner Mongolia, Heilongjiang, Chongqing, Yunnan (5)
2013	Beijing, Shanghai, Jiangsu, Zhejiang, Fujian, Henan, Guangdong, Sichuan (8)	Liaoning, Shandong, Hubei, Hunan, Tibet (5)	Hebei, Shanxi, Jilin, Anhui, Jiangxi, Guangxi, Hainan, Guizhou, Yunnan, Gansu, Qinghai, Ningxia, Xinjiang (13)	Tianjin, Inner Mongolia, Heilongjiang, Chongqing, Shaanxi (5)
2016	Beijing, Liaoning, Shanghai, Jiangsu, Zhejiang, Fujian, Henan, Guangdong, Sichuan (9)	Hebei, Shandong, Hubei, Hunan, Tibet (5)	Jilin, Anhui, Jiangxi, Guangxi, Hainan, Guizhou, Yunnan, Shaanxi, Gansu, Qinghai, Ningxia, Xinjiang (12)	Tianjin, Shanxi, Inner Mongolia, Heilongjiang, Chongqing (5)
2019	Beijing, Shanghai, Jiangsu, Zhejiang, Fujian, Henan, Guangdong, Sichuan (8)	Hebei, Anhui, Shandong, Hubei, Hunan (5)	Jilin, Heilongjiang, Jiangxi, Guangxi, Hainan, Guizhou, Yunnan, Tibet, Shaanxi, Gansu, Qinghai, Ningxia, Xinjiang (13)	Tianjin, Shanxi, Inner Mongolia, Liaoning, Chongqing (5)

**Figure 4:**
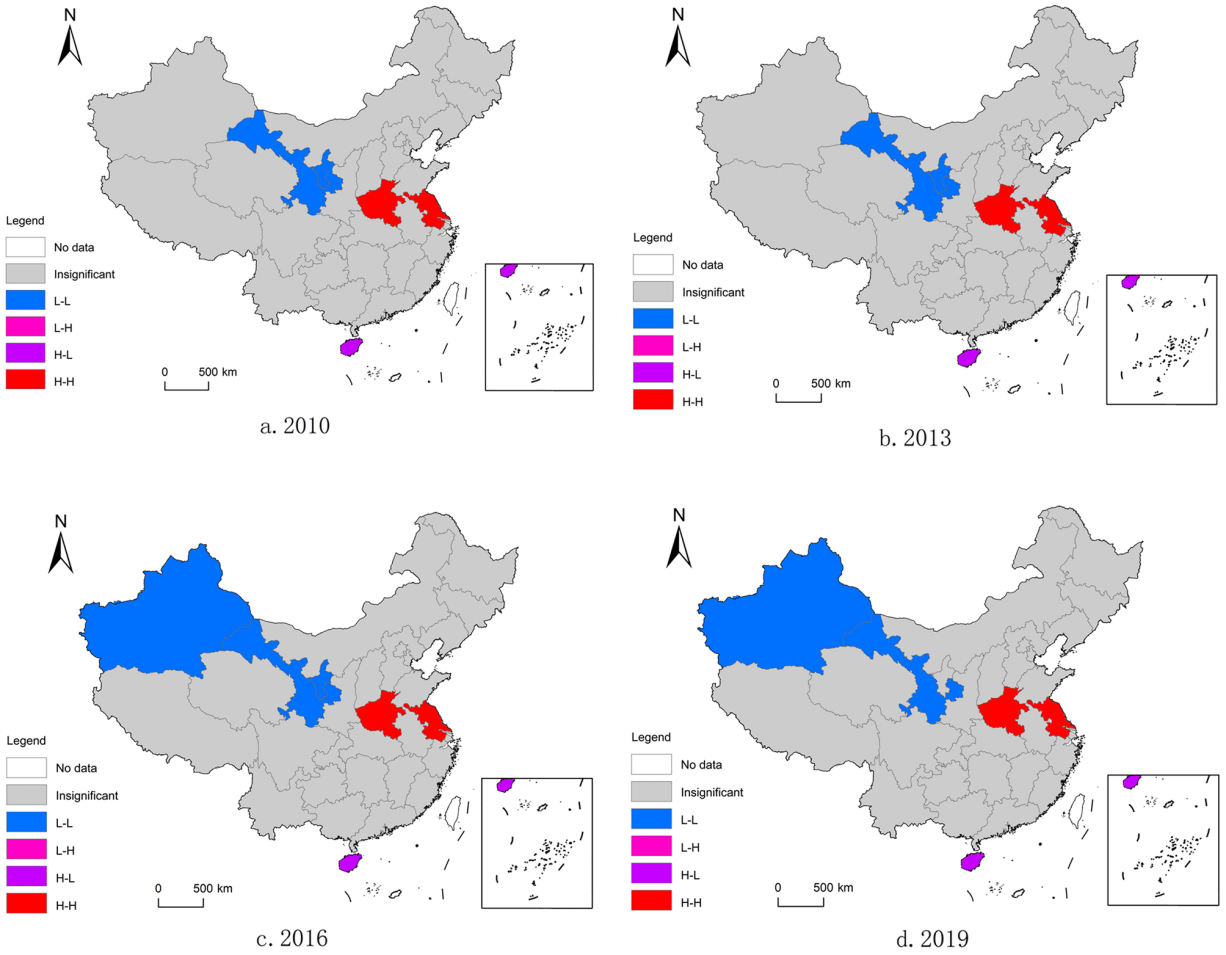
Local autocorrelation LISA plot of China’s health tourism development index (some years). *Note*: This map is based on the standard map of the standard map service system of the Ministry of Natural Resources of China (review number: GS (2019) 1822), and the base map is unmodified.

From the statistics of the table, H–H agglomeration is mainly concentrated in the eastern coastal region, L–L agglomeration is mainly concentrated in western China, and the distribution of H–L agglomeration and L–H agglomeration is more scattered, among which, H–L agglomeration is more prominent in provinces such as Tianjin, Inner Mongolia, and Chongqing, and L–H agglomeration is more prominent in provinces such as Shandong, Hubei, and Hunan. The LISA clustering map is still necessary for verification because the Moran scatter plot can only give us a preliminary assessment of the general state of spatial agglomeration. The LISA clustering map of the health tourism development index in 31 Chinese provinces from 2010 to 2019 was created using ArcGIS software (Fig. 4).

As can be seen from Fig. 4, the number of provinces where the local spatial autocorrelations of health tourism development pass the significance test is substantially less than the number listed in Table 5. H–L agglomeration, L–H agglomeration, and L-L agglomeration are the three main types of spatial agglomeration that were present in 2010, 2013, 2016, and 2019 respectively. Overall, homogeneous characteristics predominate in the spatial association pattern of health tourism development in China over the four-time transects and are complemented by heterogeneous characteristics; local spatial non-association is particularly noticeable. Specifically, Jiangsu, Henan, and other provinces and regions have always been the hot spot of health tourism development, while Gansu Province has always been the cold spot of health tourism development. The above analysis shows that although the development index of China’s health tourism is constantly improving, there is still a large distance to reach the ideal level of development.

### Dynamic evolution

The development of health tourism in China is not independent of each other in terms of geography, and the development of health tourism in each province is influenced by the region in which it is located, with strong spatial agglomeration and spatial interaction effects, as reflected in the previous analysis on the spatial-temporal pattern. Additionally, the spatial Markov chain method can be used to determine the impact of various neighborhood types on the likelihood of shifting provincial health tourism types, providing a clear picture of the spatial dynamic evolution. Table 6 presents the results of the spatial Markov transfer probability significance test for health tourism development in China. The results once more demonstrate the existence of spatial effects in this process. Therefore, based on the constructed spatial weight matrix and with the help of spatial Markov chain analysis method to further quantify this spatial effect, a comparative analysis of the dynamic evolutionary characteristics of health tourism development is conducted to explore the dynamic evolutionary process of China’s health tourism in the geographical and economic context.

**Table 6:** Significance test results for the spatial markov transfer probability *x*^2^.

Duration (year)	Q-value	Degree of freedom	*x*^2^ threshold	*P*-value
1	32.174	2	5.991	0

The measurement results of the spatial Markov chain are given in Table 7. Firstly, under the condition of considering spatial lag, the stability probabilities are 96.8%, 88.2%, 85.0%, and 25.0% for type 1, 76.9%, 91.7%, 45.5%, and 75.0% for type 2, 44.4%, 89.5%, 75.9%, and 80.0% for type 3, and type 4, respectively were 100%, 100%, 91.7%, and 100%. When the neighboring spatial lag type is 4, the stability probability of type 1 drops sharply from 96.8 to 25.0%, indicating that the provinces with low development index may break through the current development dilemma through their development under the positive “spillover effect” of the provinces with high development index. Secondly, the probability of type 1 with low development index moving to type 2 with a low to medium level under four domain types is 3.2%, 11.8%, 15.0%, and 75%, respectively, which also indicates that the proximity to provinces with high development index is conducive to improving the health tourism development index of this province and region, i.e., there is a positive “spillover effect” in geographic space. When the neighboring spatial lag type decreases from high development index type to medium–high development index type, the probability of type 3 shifting downward increases from 0 to 3.4%, and the probability of type 4 shifting downward increases from 0 to 8.3%, indicating that neighboring with provinces with lower health tourism development index will have certain negative effects on the health tourism development of this province, i.e., there is a geospatial negative “spillover effect”. The “the better is better, the worse is worse” polarization tendency is presented as a result of the “Matthew effect” characteristics of the evolution of China’s health tourism development index distribution. The analysis presented above demonstrates that the transfer of each type of development index is not geographically isolated but frequently influenced by the development of health tourism in nearby areas.

**Table 7:** Spatial Markov shift probability matrix of health tourism development types in China from 2010 to 2019.

Neighborhood type	t/t + 1	n	1	2	3	4
1	1	33	0.968	0.032	0	0
	2	13	0	0.769	0.231	0
	3	7	0	0	0.444	0.556
	4	11	0	0	0	1
2	1	20	0.882	0.118	0	0
	2	32	0	0.917	0.083	0
	3	23	0	0	0.895	0.105
	4	5	0	0	0	1
3	1	10	0.850	0.150	0	0
	2	23	0	0.455	0.545	0
	3	30	0	0.034	0.759	0.207
	4	23	0	0	0.083	0.917
4	1	10	0.250	0.750	0	0
	2	9	0	0.750	0.250	0
	3	14	0	0	0.800	0.200
	4	16	0	0	0	1

1: low development index type; 2: medium–low development index type; 3: medium–high development index type; 4: high development index type.

In summary, the spatial Markov transfer probability matrix shows that the probability values that are not immediately adjacent to the diagonal values are nearly all zero, indicating that it is unlikely, based on taking span into account, that the health tourism development index of each province will achieve leapfrogging, regardless of the index of the neighboring provinces’ health tourism development. There is no leapfrogging and the transfer of health tourism development index types only happens between adjacent rank types. The type of spatial lag in nearby areas tends to have an impact on how China’s health tourism development index evolves, but under the traditional rough tourism development model, China’s health tourism development has a strong path dependency, making it challenging to achieve short-term leapfrogging within a year. The evolution of China’s health tourism development index shows a partial “Matthew effect”, therefore, it will show a certain clustering distribution pattern in geographic space.

### Influencing factors

#### Selection of influencing factors

**Figure 5:**
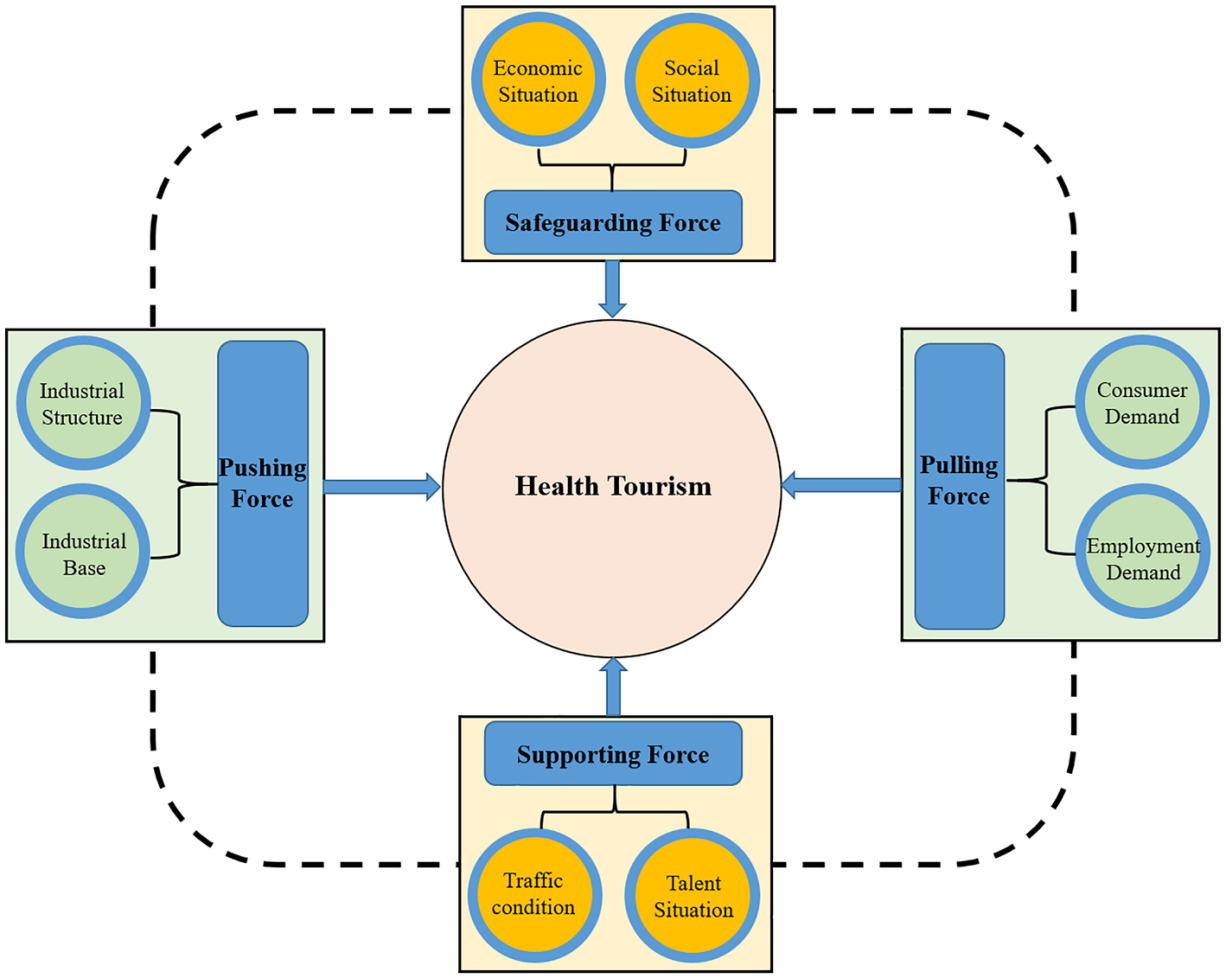
Analytical framework of factors influencing the development of health tourism.

The development of health tourism is affected by multiple factors, such as resource status, market demand, traffic condition, economic level and tourism investment, which are important driving forces for the development of health tourism in China^23,55^. Based on this reference to related literature, eight factors such as economic situation, social situation, traffic condition, talent situation, industrial structure, industrial base, consumer demand and employment demand are selected as the explanatory variables for the four major driving forces of health tourism development such as safeguarding force, supporting force, pushing force and pulling force^29,56–58^, and the analytical framework of the influencing factors of health tourism development is shown in Fig. 5. Specifically, the higher the per capita GDP, the more developed the regional economy, so the per capita GDP is chosen to measure the economic situation. The higher the level of urbanization of the population, the higher the level of population literacy, the more harmonious social atmosphere, so the level of urbanization of the population is used to measure the level of social development. The accessibility of transportation in the development of health tourism is an important support, so the per capita area of the city road is used to illustrate the traffic condition. The more students in higher education, the more favorable it is to cultivate more professionals for the development of health tourism, so the average number of students in higher education per 100,000 people was chosen to represent the talent situation. The tertiary industry can shape a good environment for the development of health tourism, so the proportion of tertiary industry in GDP is chosen to indicate the industrial structure. The state of natural resources and medical conditions is an important foundation for the development of the health tourism industry different from that of the general tourism industry, so the sum of the scores of per capita water resources and the number of practicing (assistant) physicians per 10,000 people are chosen to represent the industrial base. The higher the consumer demand for health tourism and the more employment it absorbs, the more it can stimulate the development of health tourism, so the share of domestic tourism revenue in GDP and the number of people employed in the culture, sports and entertainment industry were chosen to assess the consumer demand and employment demand respectively.

#### Analysis of influencing factors

According to Moran’s *I* index, we find a significant spatial correlation between the health tourism development of Chinese provinces. In order to further explore the influencing factors of health tourism development and its spatial effects, the spatial econometric model was chosen for the follow-up study. The test results of specific model selection are shown in Table 8.

**Table 8:** Test results of spatial econometric model selection.

Test items	Test methods	Geo-economic adjacency matrix	
		Statistics	*P*-value
SAR and SEM tests	LM-lag test	5.208	0.022
	R-LM-lag test	14.253	0.000
	LM-err test	0.769	0.380
	R-LM-err test	9.814	0.002
Fixed effect tests of SDM	SFE-LR test	19.19	0.936
	TFE-LR test	559.05	0.000
Hausman test of SDM	Hausman test	38.37	0.002
	Wald-lag test	20.73	0.008
Simplified tests of SDM	LR-lag test	17.86	0.0223
	Wald-err test	20.78	0.008
	LR-err test	18.21	0.020

From the test results in the table above, SDM with spatial fixed effect is more suitable. For comparison, SAR and SEM were also estimated and tested as well and the results are presented in Table 9. From the estimation results, $\sigma^{2}$ is significant at the 1% significance level, indicating a good fit of SDM.

**Table 9:** Estimation results of spatial econometric model.

Variables	SAR	SEM	SDM
ln*ES*	0.024**	0.028***	0.017
	(2.284)	(3.193)	(1.116)
*SS*	0.283***	0.290***	0.253***
	(3.923)	(4.043)	(3.175)
ln*TC*	0.035***	0.036***	0.038***
	(3.950)	(4.054)	(4.226)
ln*TS*	0.045***	0.042***	0.058***
	(3.006)	(2.944)	(3.717)
*IS*	0.069***	0.072***	0.062***
	(3.073)	(3.292)	(2.625)
ln*IB*	0.038***	0.037***	0.036***
	(3.747)	(3.608)	(3.455)
*CD*	0.079***	0.081***	0.074***
	(4.091)	(4.263)	(3.619)
ln*ED*	0.020**	0.020**	0.021**
	(2.142)	(2.118)	(2.299)
W*ln*ES*			0.017
			(0.782)
W*SS			− 0.188
			(− 0.892)
W*ln*TC*			− 0.027
			(− 1.024)
W*ln*TS*			− 0.038
			(− 0.906)
W*IS			0.147**
			(2.365)
W*ln*IB*			0.051**
			(2.010)
W*CD			− 0.027
			(− 0.592)
W*ln*ED*			− 0.020
			(− 1.224)
$\rho$ (Spatial lag term)	0.038		0.001
	(0.658)		(0.008)
$\lambda$ (Spatial error term)		0.026	
		(0.290)	
$\sigma^{2}$	0.0002***	0.0002***	0.0001***
	(12.449)	(12.449)	(12.450)
Log-Likelihood	920.6543	920.4804	929.5830
Observations	310	310	310
R^2^	0.530	0.526	0.540

*, **, and *** are significant at the significance level of 10%, 5%, and 1%, respectively. The "t "statistic is in parentheses.

In order to accurately judge the influencing factors and their spatial spillover effects on the development of health tourism in China, the estimation results of the direct, indirect and total effects are summarized in Table 10, where the direct effect mainly reflects the influence of the influencing factors on the development of health tourism in the region, the indirect effect mainly reflects the spatial spillover effects of the influencing factors on the development of health tourism in neighboring regions, and the total effect is the sum of the direct and indirect effects.

**Table 10:** Direct, indirect, and total effects of explanatory variables.

Variables	China			Eastern China			Central China			Western China		
	Direct effect	Indirect effect	Total effect	Direct effect	Indirect effect	Total effect	Direct effect	Indirect effect	Total effect	Direct effect	Indirect effect	Total effect
ln*ES*	0.017	0.017	0.034*	0.013	0.083***	0.096***	0.059***	− 0.004	0.055*	0.035	− 0.030	0.005
	(1.128)	(0.806)	(1.805)	(0.566)	(3.016)	(5.717)	(5.092)	(− 0.173)	(1.747)	(1.108)	(− 0.648)	(0.101)
*SS*	0.251***	− 0.184	0.067	0.249**	− 0.101	0.148	0.418***	− 0.361	0.057	0.364*	1.175**	1.539***
	(3.283)	(− 0.889)	(0.289)	(2.486)	(− 0.631)	(0.862)	(4.815)	(− 1.199)	(0.157)	(1.879)	(2.355)	(2.762)
ln*TC*	0.038***	− 0.029	0.009	0.022	− 0.037*	− 0.014	− 0.011	− 0.043	− 0.054	0.098***	0.006	0.104**
	(4.030)	(− 1.104)	(0.317)	(1.452)	(− 1.722)	(− 0.848)	(− 0.649)	(− 0.802)	(− 0.819)	(6.597)	(0.146)	(2.444)
ln*TS*	0.057***	− 0.036	0.021	0.145***	− 0.038	0.107**	0.009	0.050	0.059	− 0.067**	− 0.137	− 0.204**
	(3.604)	(− 0.792)	(0.472)	(6.227)	(− 0.727)	(2.233)	(0.670)	(1.243)	(1.222)	(− 2.031)	(− 1.641)	(− 2.271)
*IS*	0.061***	0.150**	0.211***	0.016	0.057	0.073	0.070***	0.197***	0.267***	0.037	− 0.010	0.027
	(2.654)	(2.533)	(3.310)	(0.355)	(0.963)	(1.290)	(3.559)	(2.803)	(3.101)	(0.826)	(− 0.083)	(0.182)
ln*IB*	0.037***	0.049**	0.086***	0.037***	0.034**	0.071***	0.007	0.035	0.042	0.027	0.018	0.045
	(3.725)	(2.023)	(3.337)	(3.129)	(2.276)	(5.765)	(0.628)	(1.344)	(1.372)	(1.070)	(0.312)	(0.673)
*CD*	0.075***	− 0.025	0.050	− 0.091	− 0.090	− 0.181	0.107***	− 0.061	0.046	0.144***	− 0.377***	− 0.232*
	(3.544)	(− 0.543)	(1.013)	(− 1.308)	(− 0.911)	(− 1.449)	(6.558)	(− 1.050)	(0.744)	(3.408)	(− − 3.298)	(− 1.867)
ln*ED*	0.021**	− 0.019	0.002	0.036**	− 0.106***	− 0.070***	0.003	− 0.019	− 0.016	− 0.008	− 0.044	− 0.053
	(2.357)	(− 1.105)	(0.126)	(2.094)	(− 4.465)	(− 3.328)	(0.472)	(− 1.026)	(− 0.684)	(− 0.474)	(− 1.183)	(− 1.368)

*, **, and *** are significant at the significance level of 10%, 5%, and 1%, respectively. The "t "statistic is in parentheses.

First, in terms of direct effect. Nationally, factors such as social situation, traffic condition, talent situation, industrial structure, industrial base, consumer demand and employment demand all have a positive impact on the development of health tourism. People in areas with good social situation tend to pay more attention to physical and mental health, and are more likely to accept and support the development of health tourism. Transportation accessibility changes the breadth and depth of economic ties between health tourism-related industries in the region to a certain extent. The greater the number of professionals, the more beneficial it is to the high-quality development of health tourism. A good industrial structure can promote the comprehensive and sustainable development of health tourism. The development of health tourism emphasizes “health”, and the health industry and health resources and other industrial bases are particularly important. In addition, the higher the consumer demand and the greater the benefits, the more it can directly stimulate the growth of health tourism-related investment. The more employment population absorbed and the stronger the employment demand, the more conducive to the expansion of the scale of health tourism-related industries. In terms of subregions, the positive influencing factors in eastern China are social situation, talent situation, industrial base and employment demand; in central China, the positive influencing factors are economic situation, social situation, industrial structure and consumer demand; and in western China, the positive influencing factors are social situation, traffic condition, talent situation and consumer demand. The difference in the influencing factors for the development of health tourism in the three regions also reflects the difference in the development of these regions in many areas, including the economy, society and culture. For example, the factors that have a greater impact on the development of health tourism in western China are the lower level of social development, the more inconvenient traffic condition and the lack of talent reserves, which cause the direct effect of these factors on the development of health tourism in the western region to be more significant.

Second, the indirect effect, i.e., the spatial spillover effect aspect. For the whole country, the positive spatial spillover effect of industrial structure on the development of health tourism in neighboring regions is the highest, and the indirect effect of industrial structure is greater than the direct effect. Liu &Tang (2022) pointed out that the industrial structure can characterize the level of specialization of regional tourism to a certain extent, which affects the efficiency of its resource utilization and economic benefits^59^. A good industrial structure not only promotes tourism development within the region, but also drives the improvement of tourism resource utilization efficiency and tourism specialization level in neighboring regions to a certain extent. The spatial spillover effect of the industrial base on the development of health tourism in the neighboring regions is lower than that of the industrial structure, a phenomenon that may be explained by the relatively limited radiation of natural and human resources of health tourism to the neighboring regions and the strong territoriality of these resources. Moreover, the indirect effect of the six influencing factors, including economic situation, social situation, traffic condition, talent situation, consumer demand and employment demand, do not pass the significance test. In terms of subregions, the influencing factors with spatial spillover effect in eastern China are economic situation, industrial base, traffic condition and employment demand, the influencing factors with spatial spillover effect in central China are industrial structure, and the influencing factors with spatial spillover effect in western China are economic situation and consumer demand. As mentioned above, there are reasons behind the difference in the spatial spillover effect of the factors influencing the development of health tourism in the three major regions. For example, the overall economy of western China is relatively backward, and the enhancement of its economic situation can also promote the development of health tourism in neighboring regions, and as western China further strengthens its advantages in resources, it is inevitable that it will also weaken the attractiveness of the health tourism in the neighboring regions.

Third, the total effect, i.e. the sum of direct and indirect effects. For the whole country, the total effect of industrial structure is the highest, followed by industrial base, economic situation, and the five factors of social situation, traffic condition, talent situation, consumer demand and employment demand are not significant. In terms of subregions, the factors influencing the total effect in eastern China are economic situation, talent situation, industrial structure, consumer demand and employment demand, the factors influencing the total effect in central China are economic situation and industrial structure, and the factors influencing the total effect in western China are social situation, traffic condition, talent situation and consumer demand.

Finally, in order to avoid endogeneity problems between the data, the spatial weight matrix of economic adjacency was applied instead of the geo-economic adjacency matrix to test the robustness of the factors influencing the development of health tourism (Table 11). The results show that the benchmark regression results remain unchanged, indicating that the analysis is robust.

**Table 11:** Estimation results of robustness test.

Variables	Direct effect	Indirect effect	Total effect
ln*ES*	0.019	0.015	0.034*
	(1.196)	(0.736)	(1.739)
*SS*	0.233***	− 0.167	0.066
	(2.988)	(− 0.811)	(0.279)
ln*TC*	0.037***	− 0.020	0.017
	(3.969)	(− 0.740)	(0.605)
ln*TS*	0.056***	− 0.041	0.015
	(3.515)	(− 0.906)	(0.327)
*IS*	0.060***	0.150**	0.210***
	(2.589)	(2.489)	(3.222)
ln*IB*	0.039***	0.043*	0.082***
	(3.953)	(1.757)	(3.029)
*CD*	0.076***	− 0.022	0.054
	(3.552)	(− 0.481)	(1.074)
ln*ED*	0.020**	− 0.017	0.004
	(2.259)	(− 0.975)	(0.198)

*, **, and *** are significant at the significance level of 10%, 5%, and 1%, respectively. The "t "statistic is in parentheses.

## Conclusion and discussion

### Conclusion

This paper empirically analyzes the development of health tourism based on the data of 31 provinces in China from 2010 to 2019, using various research methods. Firstly, the index system for measuring the health tourism development index is constructed by reading related books and literature, and then the entropy method, Thiel index, exploratory spatial data analysis, spatial Markov chain and spatial econometric model are fully utilized to comprehensively explore China's health tourism development focusing on several aspects such as the difference status of the development of health tourism, spatial pattern, dynamic evolution and influencing factors. The main conclusions and findings are as follows:

1. Health tourism development index and its change: China's health tourism development index increased from 0.12 to 0.23, with a growth rate of 7.67%, a strong development momentum. However, the current development index remains relatively low, highlighting the need for necessary measures to promote the development of health tourism. In addition, the development index of eastern China is higher than that of central and western China, while the growth rate of western China is higher than that of central and eastern China, reflecting a possible “catching-up effect” between regions.
2. Difference status of health tourism development: The intra-regional differences in China’s health tourism development have always been higher than the level of inter-regional difference, and the difference between the two has remained relatively stable. In addition, the overall difference in western China from 2010 to 2012 was greater than that in eastern China and central China, and after 2012 the overall difference in eastern China was always greater than that in western China and central China, and the overall difference in central China was always the lowest.
3. Spatial-temporal pattern of health tourism development: There is a positive correlation in the global space, and the local space shows a clustering trend. Across the four time cross-sections of 2010, 2013, 2016 and 2019, Jiangsu, Henan and other provinces and cities have always been the hotspot areas of health tourism development, and Gansu Province has been the cold spot area of health tourism development.
4. Dynamic evolution of health tourism development: There is a certain “Matthew effect” in geographic space, and the spatial spillover effect is obvious. Meanwhile, there is heterogeneity in the spatial spillover effect in different regional contexts, which in general. At the same time, the spatial spillover effect in different regional contexts is heterogeneous, and it maintains a “stable” state, which is not conducive to the further development of health tourism.
5. Health tourism development influencing factors: As far as the development of health tourism in China as a whole is concerned, factors such as social situation, traffic condition, talent situation, industrial structure, industrial base, consumer demand and employment demand have positive direct effect, Additionally, industrial structure and industrial base have a positive indirect effect, while industrial structure, industrial base, and economic situation exert a positive total effect.

### Discussion

Some studies have already shown that the COVID-19 pandemic has had a significant impact on health and wellness tourism and that the health tourism market during the COVID-19 pandemic is in crisis^22^. However, the study period of health tourism in this paper is mainly from 2010 to 2019 before the COVID-19 pandemic, and there is little clarity on how health tourism changed during and after the COVID-19 pandemic, so it is necessary to explore it properly. While the tourism industry, which has a highly mobile population, strong industry linkages, and high sensitivity of its own, has been hit hard by COVID-19, even leading to a global crisis for the tourism and hospitality sectors^60^, it has at the same time presented important opportunities for the future development of tourism. During the COVID-19 pandemic, people’s perceptions of the COVID-19 pandemic event itself^61^ and the perceived risks^62^ weakened their willingness to travel in several ways. After the COVID-19 pandemic, people would pay more attention to the risk information and they could join health tourism in the post-pandemic period to enhance their personal physical and mental health^25,63^. It has also been suggested that organizations offer personalized health tourism packages to their employees accordingly, invoking a sense of Perceived Organizational Support (POS) among them^64^. In conclusion, the COVID-19 pandemic has led to an increase in consumers’ awareness of self-protection and safety and hygiene, changes in travel psychology and demand, and the promotion of a new health tourism industry^65^. At the same time, reflection on the COVID-19 pandemic has cultivated a new generation of tourism consumers to establish a new concept of tourism, and the pursuit of a healthy lifestyle by tourism consumers after the COVID-19 pandemic is conducive to the rapid development of health tourism^66^. Then, in order to promote the sustainable development of health tourism in the post-pandemic era, this study puts forward the following policy recommendations based on the analysis above:

1. Choosing a practical development model. By analyzing the factors influencing the development of health tourism in China, eastern China, central China and western China, it can be found that there are significant differences in the foundation and conditions for the development of health tourism in each region. Therefore, each province in China should choose the development mode of health tourism according to its industrial reality. For example, provinces with good resource endowment should focus on the development mode of health tourism characteristics, and provinces with good economic level and market foundation should focus on the capital-driven mode of health tourism. In short, the key point of choosing different development modes is to combine local advantages with the development of health tourism. Achieving the integration and innovative growth of the health tourism industry necessitates establishing a pattern of “multi-party participation, multi-investment, and mutual win-win situation”.
2. Constructing a complete industrial ecosystem. When constructing the health tourism evaluation index system in the previous section, it has been made clear that there are many health tourism-related industries, and they are roughly characterized by resource contribution, function extension, technology and service penetration among each other. Therefore, it is necessary to promote the formation of the health tourism industrial ecosystem through the extension and integration of industries, the deepening of industrial synergy and the enhancement of industrial association. One is to build a health tourism industry system centered on the core health tourism industry and supported by related industries^67^. Secondly, the value aggregation effect will be realized through resource sharing and function complementation among industries. Thirdly, the government should increase investment in infrastructure and talent training to strengthen the foundation and support for the development of health tourism.
3. Coordination between market mechanism and government behavior. The government and the market are the “two hands” that regulate the development of health tourism, and they complement each other and cooperate. The organic combination of these “two hands” not only ensures the correct direction of macro-industry development strategy, but also realizes the efficient allocation of micro-market resources^68^. We should build a “government-guided, market-led, multi-party participation” “multi-body, multi-level” health tourism external guarantee system based on the guiding ideology of “resource integration, complementary advantages and mutual benefits”.
4. Create a model district for the integrated development of health tourism. As already analyzed in the previous section, health tourism has the outstanding characteristics of integrated development, and at the same time, the development of health tourism needs to be led and driven, which is an important driving force to promote the development of health tourism. Therefore, it is necessary to actively give some leading health tourism enterprises with strength and potential sufficient regional policy preferences and financial support, and actively play the economic demonstration role of leading enterprises, model districts, and excellent enterprises in tourism integration and development, so as to enhance the motivation of small and medium-sized tourism and health-related enterprises that are still on the sidelines to carry out cooperative development. Furthermore, small and medium-sized health tourism enterprises that are new to the health tourism integrated development system should be provided with necessary guidance in various ways^49^.
5. Enhancing the service experience of health tourism. The essence of tourism is still a service industry, which inevitably cannot be separated from the high demand for service experience. Therefore, it is not only necessary to make more efforts in the design of health tourism products and explore better products and service forms to enhance the tourism experience^53^, but also to optimize the comprehensive management capacity of health tourism bases^69^. The first is to build a intelligent management platform for health tourism bases to strengthen the digital management capacity. The second is to adopt the system of integral feedback and tracking survey to strengthen the capacity of base operation and management mode innovation. The third is to strengthen regional ties, build embedded and multi-functional health tourism service organizations, revitalize idle resources and strengthen the capacity of resource conversion innovation.
6. Actively developing the international market. Health tourism has a wide range of international markets, and the development of health tourism must not ignore this part of the market. The development of international health tourism is very rapid, and while China is a late starter, in the international tourism market more plays the role of the source^70^. China’s health tourism industry should give full play to its advantages in participating in international competition, enhance the international popularity of China’s health tourism through publicity and promotion, and strengthen its international influence, and at the same time, establish an all-round, multi-level sales channel for health tourism products and services^71^, and actively expand the international market through multiple measures.

Admittedly, this study does have some flaws, including the following: The construction of the index system still needs improvement due to the complexity, comprehensiveness, and specificity of health tourism itself. The improvement and spatial optimization of the health tourism development index is controlled by multiple factors, and the discussion of the influence of multiple factors on its development is still insufficient. The entropy method, exploratory spatial data analysis method, spatial Markov chain and spatial econometric model have their limitations, which may cause some calculation errors and deviate from the actual situation of the research subjects. Finally, in future research, we can also obtain city and county-level data to conduct more comprehensive and in-depth research and pay attention to the new paths and directions of health tourism development in the post-epidemic era.

## Data Availability

The data used to support the findings of this study are available from the corresponding author upon request.
